# Antagonism of CGRP Receptor: Central and Peripheral Mechanisms and Mediators in an Animal Model of Chronic Migraine

**DOI:** 10.3390/cells11193092

**Published:** 2022-09-30

**Authors:** Rosaria Greco, Chiara Demartini, Miriam Francavilla, Anna Maria Zanaboni, Cristina Tassorelli

**Affiliations:** 1 Unit of Translational Neurovascular Research, IRCCS Mondino Foundation, 27100 Pavia, Italy; 2 Department of Brain and Behavioral Sciences, University of Pavia, 27100 Pavia, Italy

**Keywords:** olcegepant, chronic migraine, inflammation

## Abstract

Calcitonin-gene-related peptide (CGRP) plays a key role in migraine pathophysiology and more specifically in the mechanisms underlying peripheral and central sensitization. Here, we explored the interaction of CGRP with other pain mediators relevant for neuronal sensitization in an animal model of chronic migraine. Male Sprague-Dawley rats were exposed to nitroglycerin (NTG, 5 mg/kg, i.p.) or vehicle co-administered with the CGRP receptor antagonist olcegepant (2 mg/kg i.p.), or its vehicle, every other day over a 9-day period. Twenty-four hours after the last injection of NTG (or vehicle), behavioral test and ex vivo analysis were performed. Olcegepant attenuated NTG-induced trigeminal hyperalgesia in the second phase of the orofacial formalin test. Interestingly, it also reduced gene expression and protein levels of CGRP, pro-inflammatory cytokines, inflammatory-associated miRNAs (miR-155-5p, miR-382-5p, and miR-34a-5p), and transient receptor potential ankyrin channels in the medulla–pons area, cervical spinal cord, and trigeminal ganglia. Similarly, olcegepant reduced the NTG-induced increase in CGRP and inflammatory cytokines in serum. The findings show that the activation of the CGRP pathway in a migraine animal model was associated to the persistent activation of inflammatory pathways, which was paralleled by a condition of hyperalgesia. These molecular events are relevant for informing us about the mechanisms underlying chronic migraine.

## 1. Introduction

Migraine is associated with the activation of the trigeminovascular system, which induces peripheral and central sensitization [1]. The role of central sensitization, and the associated increase in the transmission of pain signals along the second and third neuron, seems particularly important for chronic migraine. 

A huge number of studies support the involvement of calcitonin gene-related peptide (CGRP) in the development of sensitization and enhanced pain sensibility that characterize migraine pain [2]. CGRP exists in two forms: α-CGRP predominates in the peripheral and central nervous system, whereas β-CGRP is mostly distributed in the enteric nervous system [3]. α-CGRP is released from trigeminal nerves during migraine attacks. The activation of the trigeminal nerve causes an antidromic release of α-CGRP to induce non-endothelium-mediated vasodilatation. In the trigeminal nucleus caudalis (TNC), CGRP acts on second-order neurons to transmit pain signals through the brainstem and midbrain to higher levels [4]. The CGRP receptors are highly distributed throughout the trigeminovascular system, particularly, but not limited to, on neurons and glial cells [5]. CGRP is also involved in mast cell degranulation and thus in neurogenic inflammation [6]. In vitro, CGRP enhances pro-inflammatory cytokine’ expression and release from rodent satellite glial cells of the trigeminal ganglia (TGs) [7], as well as from human peripheral blood mononuclear cells [8]. Circulating cytokines are known to be involved in the inflammatory pain phenomenon, and indirect evidence suggests that the release of these mediators from the trigeminovascular endings is involved in the initiation and persistence of pain [9]. The CGRP inflammatory mediator relationship may be bidirectional, since inflammatory mediators released during a migraine attack lead to the activation of transient receptor potential ankyrin 1 channels (TRPA1) [10,11], located on the trigeminal afferents, which in turn favor the release of CGRP [12,13]. The presence of inflammation in patients with migraine disease has been confirmed; for instance, several plasma cytokines, including tumor necrosis factor alpha (TNF-alpha), interleukin (IL)-1beta, and IL-6, increase during migraine attacks and in attack-free intervals [14]. Thus, the activation of the trigeminovascular system and the release of CGRP and neurogenic inflammation are key factors in migraine pathogenesis, together with the maintenance of a sensitized neuronal state [15,16,17,18]. 

Clinical studies confirmed the therapeutic potential of blocking CGRP in migraine pain by different CGRP receptor antagonists and monoclonal antibodies targeting either the CGRP or its receptor in the acute and preventive treatment of migraine with positive results [19,20,21]. Besides pain transmission, the blockade of CGRP signaling may exert beneficial effects also in relation to the inflammatory pathway in migraine, although the mechanisms are still not fully understood [22,23]. 

In the multifaceted scenario of migraine pathophysiology, there are also other elements that appear to be involved. MicroRNAs (miRNAs) are short non-coding RNAs that regulate a multiplicity of cellular processes, including immune/inflammatory responses and migraine pain [24,25]. Recently, we demonstrated that in patients with migraine disease, CGRP plasma levels and miR-382-5p and miR-34a-5p gene expression in peripheral cells are associated with each other at the individual level across the migraine spectrum [26]. In addition, CGRP plasma levels were positively correlated with the peripheral levels of both miRNAs, suggesting an interaction between CGRP and these signaling molecules. CGRP may induce IL-6 gene expression in macrophages by the upregulation of circular RNA_007893, a modulator of miR-485-5p [27]. 

The aim of this study was to explore in more depth the interplay between the neuropeptide CGRP and the inflammatory mediators within the mechanisms of neuronal sensitization in an animal model of chronic migraine, using olcegepant as a pharmacological probe. Specifically, we used the experimental animal model based on chronic systemic administration of nitroglycerin (NTG) that produces cephalic and extracephalic hypersensitivity [28,29,30] and evaluated ongoing changes of signaling molecules linked to inflammation in specific areas. 

## 2. Materials and Methods

### 2.1. Animals and Experimental Design

Adult male Sprague-Dawley rats, weighing 250–270 g, were used in this study and randomly allocated in the different experimental groups, as reported in Table 1. 

The IASP’s guidelines for pain research in animals were followed [31]. All procedures were conducted in accordance with the European Convention for Care and Use of Laboratory Animals, and the experimental protocols were approved by the Italian Ministry of Health (no. 691/2020-PR). The animals were housed in the animal facility of the University of Pavia (Pavia, Italy) in groups of two per cages under controlled conditions (i.e., temperature 21–22 °C, 60–50% relative humidity) and 12/12 h light cycle (with lights on at 7.00 a.m.). Food and water were available ad libitum. Upon arrival, animals were habituated to the housing conditions for one week before the experimental testing. The experiments were performed in a randomized manner by an experimenter blinded to treatments.

NTG (Bioindustria L.I.M., Novi Ligure (AL), Italy) was prepared from a stock solution of 5.0 mg/1.5 mL dissolved in 27% alcohol and 73% propylene glycol. For injections, NTG was further diluted in saline (0.9% NaCl) to reach the final concentration of 6% alcohol and 16% propylene glycol. 

**Figure 1:**
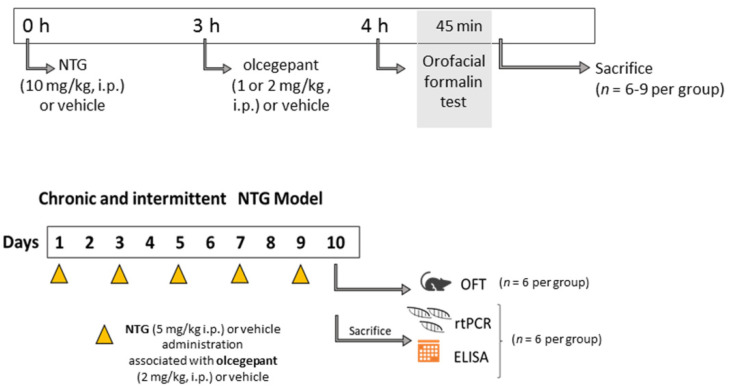
Experimental design of the acute and chronic models with specific procedures. Acute migraine model: after treatments, the animals underwent the orofacial formalin test; at the end of the behavioral test, all animals were sacrificed. Chronic migraine model: after treatments, the animals were divided into two different experimental sets. In the first set, the animals underwent the orofacial formalin test and then they were sacrificed. Within the second set, after sacrifice, the samples were collected to be used for rt-PCR and ELISA analysis. ELISA: enzyme-linked immunosorbent assay; *n*: number of animals per group; NTG: nitroglycerin; OFT: orofacial formalin test; rt-PCR: real time polymerase chain reaction.

To test the appropriate dose of olcegepant able to counteract NTG-induced trigeminal hyperalgesia, we first tested two doses of the CGRP receptor antagonist in the acute animal model of migraine on the basis of a single NTG (10 mg/kg, i.p.) administration. For this purpose, groups of 6–9 rats were treated with olcegepant (1 or 2 mg/kg, i.p.) or vehicle (PEG 200/Tween-80/saline 1:1:18, 1 mL/kg, i.p.) 3 h after NTG (10 mg/kg, i.p.) or vehicle administration. Four hours after NTG (or vehicle) injection, the animals underwent the orofacial formalin test (Figure 1). The latency of h from NTG injection and the administration timing and doses of olcegepant were selected in agreement with previous observations [28,29,32,33,34].

The optimal dose identified with the above-described set of experiments was used within the animal model of chronic migraine, where 4 groups of 6 rats each received NTG (5 mg/kg, i.p.) or vehicle injection co-administered with olcegepant 2 mg/kg i.p. or its vehicle (1 mL/kg, i.p.) every 2 days over a 9-day period (see Table 1 for experimental group assignments). 

Twenty-four hours after the last injection of NTG (or vehicle), a first set of rats underwent the orofacial formalin test. A second set of animals (not exposed to the orofacial formalin test) was used to evaluate CGRP, miRNAs, pro-inflammatory cytokines, and *TRPA1* gene expression in medulla-pons, cervical spinal cord (CSC), and TGs. In the same areas, we also evaluated the protein levels of the same cytokines. Additionally, we assayed CGRP serum levels (Table 1 and Figure 1).

### 2.2. Orofacial Formalin Test

Since NTG is systemically administered and its sensitization properties can induce cephalic and extra-cephalic changes, the orofacial formalin test was used in association with the NTG model to specifically activate the trigeminal system. Such a combination allows for the study of NTG-induced hyperalgesia at the trigeminal level, reflecting the orofacial/cephalic hypersensitivity observed in migraine patients. 

The procedures applied for the behavioral test were those extensively described elsewhere [35]. Briefly, after rats’ acclimatization (20 min) to the test chamber, they were injected subcutaneously with 50 μL of formalin 1.5% into the right upper lip. Face rubbing was measured by a researcher blind to treatments, counting the seconds the animal spent grooming the injected area with the ipsilateral forepaw or hindpaw 0–3 min (Phase I) and 12–45 min (Phase II) after formalin injection.

### 2.3. Rt-PCR and Enzyme-Linked Immunosorbent Assay (ELISA)

Rats of the second experimental set of the chronic model were euthanized under deep anesthesia (sodium thiopental) after the last NTG of vehicle injection. After decapitation, medulla–pons (bregma, −13.30 to −14.60 mm), CSC (C1–C2), and TGs were quickly dissected out, divided into right and left parts, rinsed in cold sterile 0.9% NaCl solution, placed in cryogenic tubes, and immediately frozen in liquid nitrogen. They were subsequently kept at −80 °C until rt-PCR processing for TNF-alpha, IL-1beta protein and gene expression, α-CGRP and TRPA1 gene expression, and miRNA evaluation. We selected medulla–pons and CSC because they contain several nuclei that play an important role in the mediation of migraine pain, e.g., TNC, locus coeruleus, and periacqueductal gray [36,37].

For mRNA, all procedures were performed under RNase-free conditions; after RNA extraction, the absorbance ratios (260/280 nm) ranged from 1.9 to 2.0 in all RNA samples, indicating no significant protein (including of blood origin) contamination. mRNA levels were measured by rt-PCR [38]. Primer sequences obtained from the Primer3 (https://primer3.ut.ee/, accessed on 14 January 2021) are reported in Table 2. Glyceraldehyde 3-phosphate dehydrogenase (GAPDH), whose expression remained constant in all experimental groups, was used for normalization.

The same RNA was used for miRNA extraction in the same areas. Synthesis of cDNA was performed by using MirXMirna First Strand Synthesis (Takara-Diatech Labline, Jesi-Ancona, Italy), and TB Green q-Rt PCR was used (Takara-Diatech, Labline Jesi-Ancona, Italy) to determine expression levels of miR-155-5p, miR-34a-5p, and miR-382-5p. miRNAs expression was normalized with U6 (a type of small nuclear RNA), used as a housekeeping gene. The primers of miRNAs were selected from the Primer3 software (https://primer3.ut.ee/, accessed on 26 May 2021) and synthesized by Sigma Aldrich (Milan, Italy) (Table 2). Triplicate reactions were averaged for each mRNA and miRNA. The amount of mRNA was normalized to GAPDH or U6 using the 2–ΔΔCT method.

Pro-inflammatory cytokines in medulla–pons, CSC, and TG were evaluated using the ELISA procedure. All tissues were weighed and homogenized using a Precellys homogenizer, and central and peripheral cytokine levels were analyzed using a commercial ELISA kit (Diaclone Co, Besançon, France), adhering to the manufacturer’s instructions. 

For CGRP, TNF-alpha, and IL-1beta serum levels, the blood samples were collected in a clot activator with gel separator serum tubes and centrifuged for 15 min at 1000× *g* at 2–8 °C. Protein levels were measured using commercial ELISA kits (α-CGRP: Elabsciences, Houston, TX, USA; TNF-alpha and IL-1beta: Diaclone Co, Besançon, France). The measured absorbance of the samples in a microplate reader (Biotek, Santa Clara, CA, USA) was compared with a standard curve, and the concentrations were calculated.

### 2.4. Statistical Analysis

An a priori power analysis was conducted to determine the required sample size needed to obtain a statistical power of 0.80 at an alpha level of 0.05 (GPower version 3.1.9.4, Franz Faul, University Kiel, Kiel, Germany). We hypothesized a difference in total nociceptive response in Phase II of the orofacial formalin test (face rubbing time, in the acute and the chronic models) between rats injected with NTG and rats injected with NTG + olcegepant of that at least equal to the control condition (NTG = 160 ± 14 SEM; CT = 135 ± 18 SEM), and thus we estimated a sample size of at least 6 rats in each experimental group with an effect size of 1.55. The data were tested for normality using the Shapiro–Wilk normality test and considered normal. For nociceptive responses, gene expression, and protein levels, the statistical differences between groups were determined using the one-way ANOVA followed by post hoc Tukey’s multiple comparisons test. 

A probability level of <5% was considered significant.

## 3. Results

### 3.1. Acute Migraine Model

#### Orofacial Formalin Test

**Figure 2:**
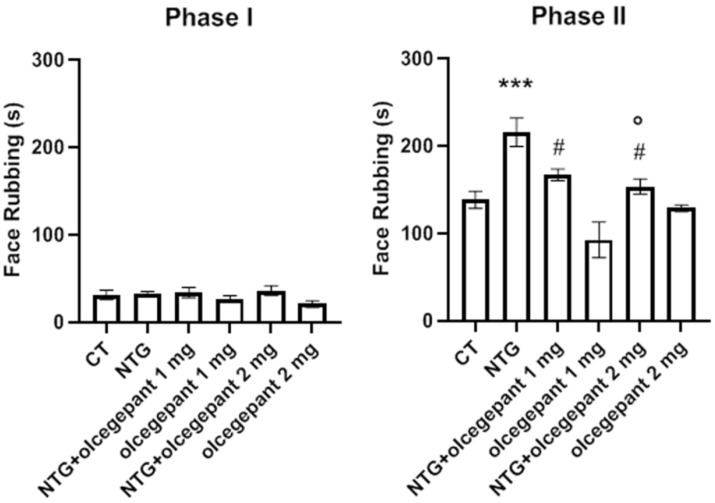
Time of face rubbing (expressed in seconds) during Phase I and II of the orofacial formalin test following acute systemic adminsitration of nitroglycerin (NTG)/vehicle and olcegepant/vehicle. Data are expressed as mean ± SEM. One-way ANOVA followed by Tukey’s multiple comparisons test: *** *p* < 0.001 vs. control (CT), olcegepant 1 mg and olcegepant 2 mg; ° *p* < 0.05 vs. NTG; # *p* < 0.05 vs. olcegepant 1 mg.

As illustrated in Figure 2, NTG administration induced a hyperalgesic state that was detectable as an increase in nocifensive behavior (total face rubbing time) during Phase II of the orofacial formalin test. Only 2 mg/kg of olcegepant significantly reduced NTG-induced nocifensive behavior in Phase II compared with NTG group, demonstrating a dose-dependent effect on trigeminal hyperalgesia. No significant effect was observed when olcegepant was injected with NTG vehicle compared with the CT group. No significant differences among groups were seen during Phase I of the test. The 2 mg/kg dose of olcegepant was then adopted to perform all the evaluations within the chronic migraine model.

### 3.2. Chronic Migraine Model

#### 3.2.1. Orofacial Formalin Test

**Figure 3:**
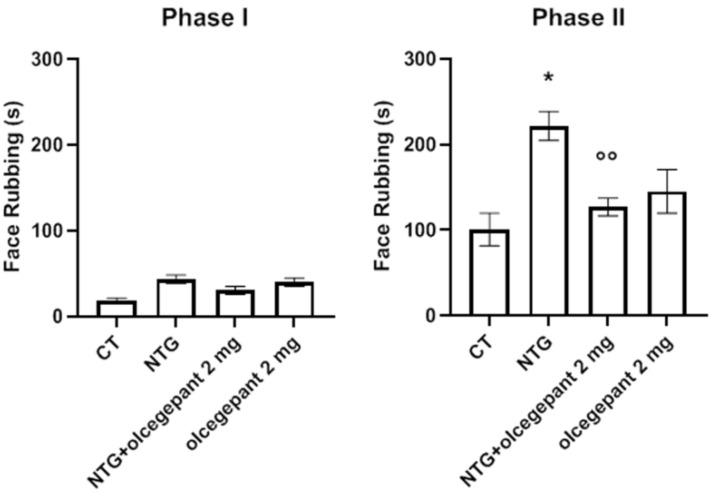
Time of face rubbing (expressed in seconds) during Phase I and II of the orofacial formalin test following chronic systemic administration of nitroglycerin (NTG)/vehicle and olcegepant/vehicle. Data are expressed as mean ± SEM. One-way ANOVA followed by Tukey’s multiple comparisons test: * *p* < 0.05 vs. control (CT) and olcegepant 2 mg; °° *p* < 0.01 vs. NTG.

As illustrated in Figure 3, NTG administration induced a persistent hyperalgesic state, which was detectable as an increase in nocifensive behavior (total face rubbing time) during Phase II of the orofacial formalin test. This hyperalgesic state was detected 24 h after the last NTG treatment. The NTG-induced increase in face rubbing time was prevented by the chronic administration of olcegepant, confirming a key role of CGRP in the central and peripheral sensitization. Olcegepant did not induce any significant change in the orofacial formalin test when administered to the rats treated with NTG vehicle. No significant differences among groups were seen during Phase I of the test.

#### 3.2.2. CGRP 

**Figure 4:**
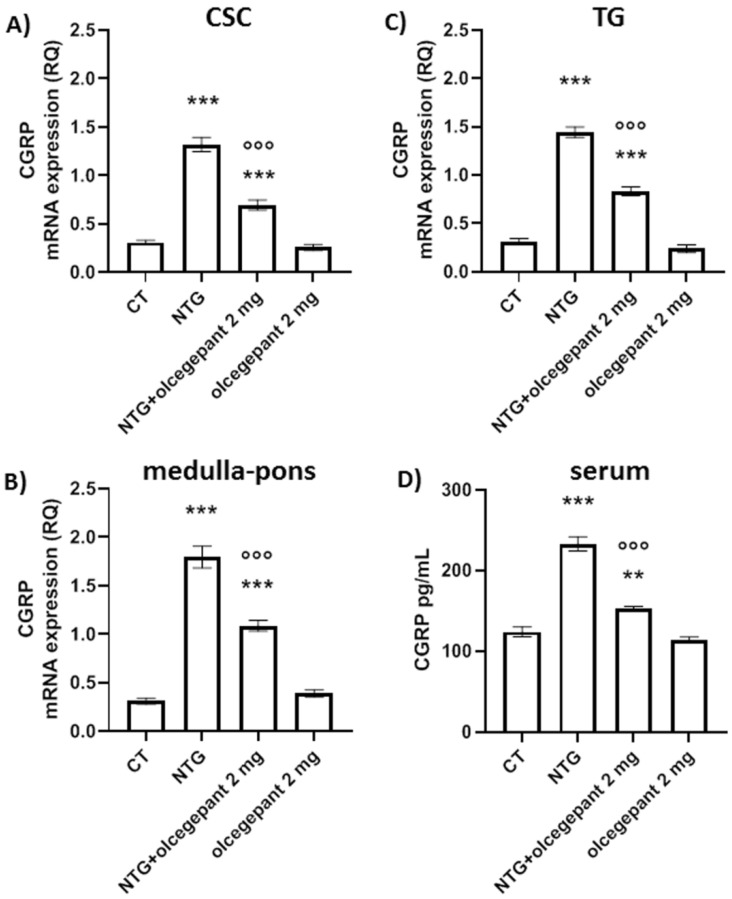
CGRP mRNA levels expressed as relative quantification (RQ) in cervical spinal cord (CSC) (**A**), medulla–pons (**B**), and trigeminal ganglia (TG) (**C**); CGRP serum levels expressed as pg/mL (**D**). Data are expressed as mean ± SEM; one-way ANOVA followed by Tukey’s multiple comparisons test. ** *p* < 0.01 and *** *p* < 0.001, vs. control (CT) and olcegepant 2 mg; °°° *p* < 0.001 vs. nitroglycerin (NTG).

Chronic NTG treatment increased CGRP gene expression in the central areas (CSC and medulla–pons, Figure 4A,B, respectively) and in the TG (Figure 4C) when compared with the control (CT) group. It also significantly increased CGRP serum levels (Figure 4D). These changes were significantly inhibited by chronic olcegepant treatment (Figure 4). No effect on CGRP gene expression and serum levels was observed when olcegepant was given with NTG vehicle (olcegepant 2 mg group).

The data suggest that olcegepant treatment reduces CGRP levels by blocking its receptor, probably via a negative feedback loop on NTG-induced inflammation.

#### 3.2.3. Cytokines 

**Figure 5:**
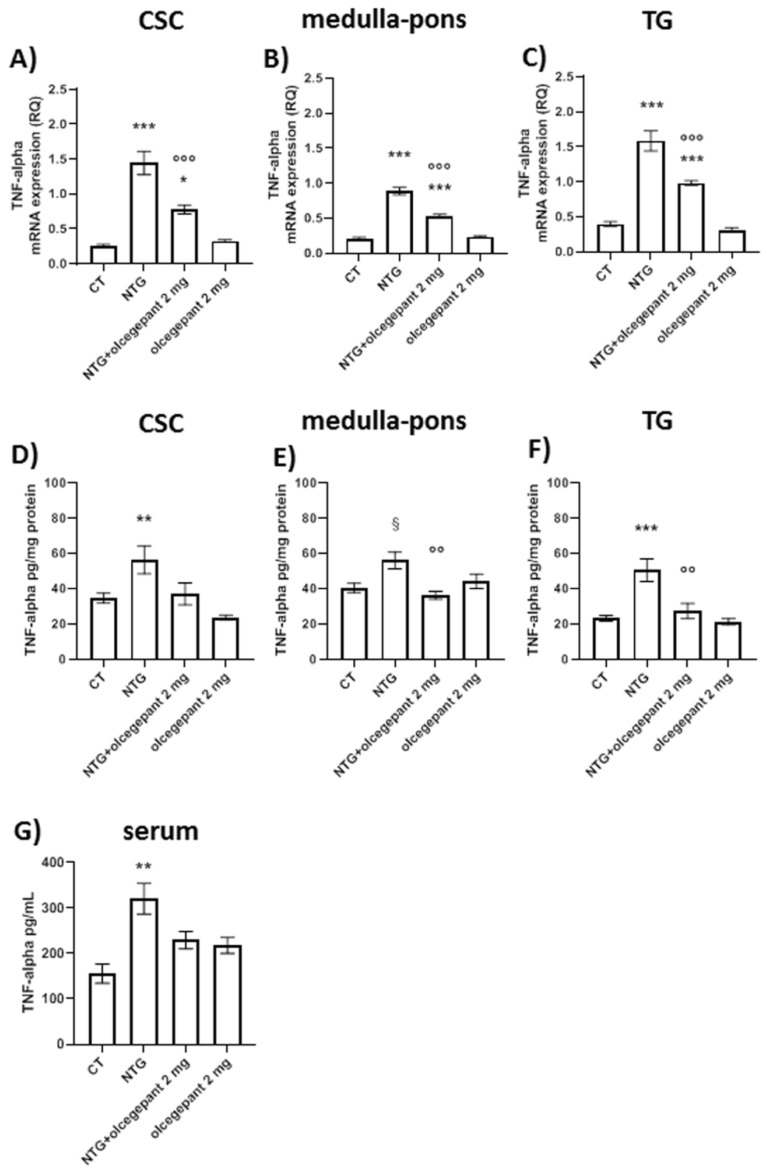
*TNF-alpha* gene expression levels (**A**–**C**) and protein levels (**D**–**F**) in cervical spinal cord (CSC), medulla–pons and trigeminal ganglion (TG), and TNF-alpha serum levels (**G**). mRNA levels are expressed as relative quantification (RQ); protein levels in tissues are expressed as pg/mg of protein and in serum are expressed as pg/mL. Data are expressed as mean ± SEM; one-way ANOVA followed by Tukey’s multiple comparisons test. * *p* < 0.05, ** *p* < 0.01, and *** *p* < 0.001 vs. control (CT) and olcegepant 2 mg; °° *p* < 0.01 and °°° *p* < 0.001 vs. nitroglycerin (NTG); § *p* < 0.05 vs. CT.

**Figure 6:**
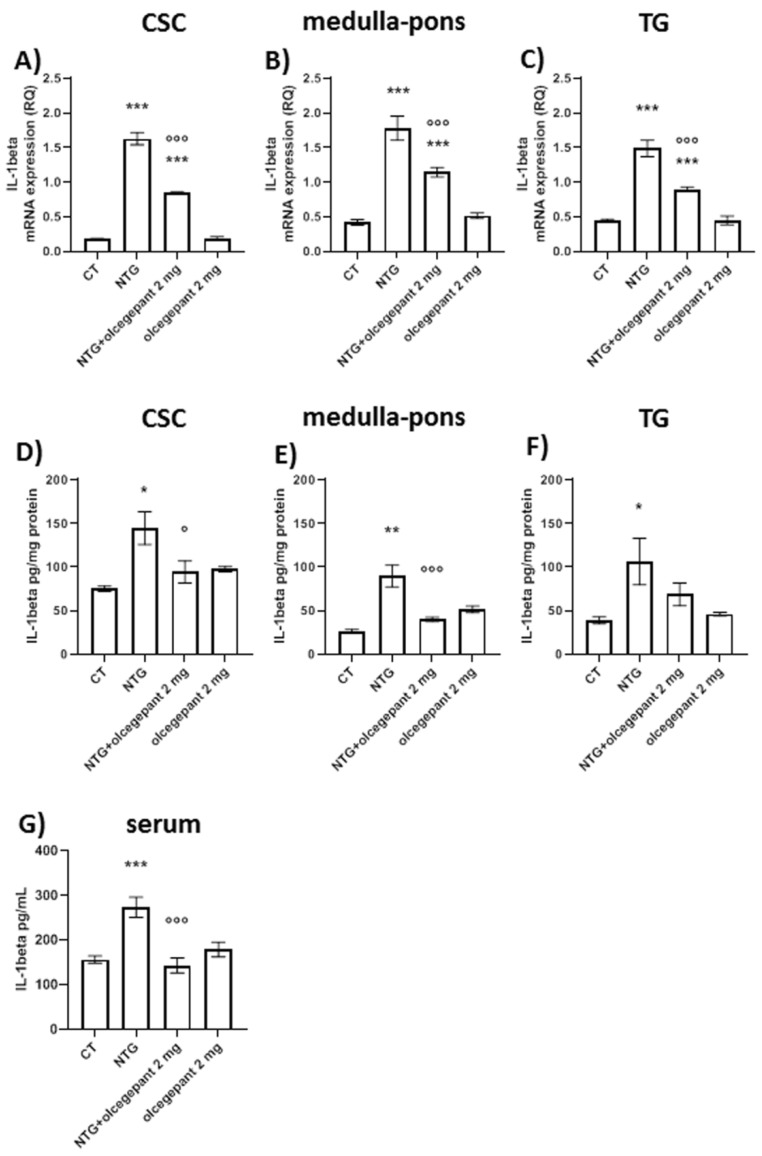
*IL-1beta* gene expression levels (**A**–**C**) and protein levels (**D**–**F**) in cervical spinal cord (CSC), medulla–pons and trigeminal ganglion (TG), and IL-1beta serum levels (**G**). mRNA levels are expressed as relative quantification (RQ); protein levels in tissues are expressed as pg/mg of protein and in serum are expressed as pg/mL. Data are expressed as mean ± SEM; one-way ANOVA followed by Tukey’s multiple comparisons test. * *p* < 0.05, ** *p* < 0.01, and *** *p* < 0.001 vs. control (CT) and olcegepant 2 mg; ° *p* < 0.05 and °°° *p* < 0.001 vs. nitroglycerin (NTG).

Chronic NTG treatment increased gene expression and protein levels of TNF-alpha (Figure 5) and IL-1-beta (Figure 6) in medulla–pons, CSC, and TG compared to the CT group; moreover, the NTG challenge significantly increased TNF-alpha (Figure 5G) and IL-1-beta (Figure 6G) serum levels compared to the CT group. Chronic treatment with olcegepant induced a significant decrease in NTG-induced mRNA TNF-alpha in all the areas under evaluation (Figure 5A–C). Olcegepant administration also reduced TNF-alpha protein levels in the medulla–pons and TG (Figure 5D–F). Olcegepant reduced the NTG-induced increase in IL-1beta mRNA in all the areas under investigation. It also reduced IL-1beta protein levels in the CSC and medulla–pons and in the serum (Figure 6A–F). Olcegepant did not induce any significant change in the parameters under evaluation when administered to the rats treated with NTG vehicle. Data are shown in Figure 5 and Figure 6.

These findings indicate that the release of cytokines is mediated by CGRP probably from the activated glial cells, which is responsible for the persistence of trigeminal pain.

#### 3.2.4. microRNAs 

**Figure 7:**
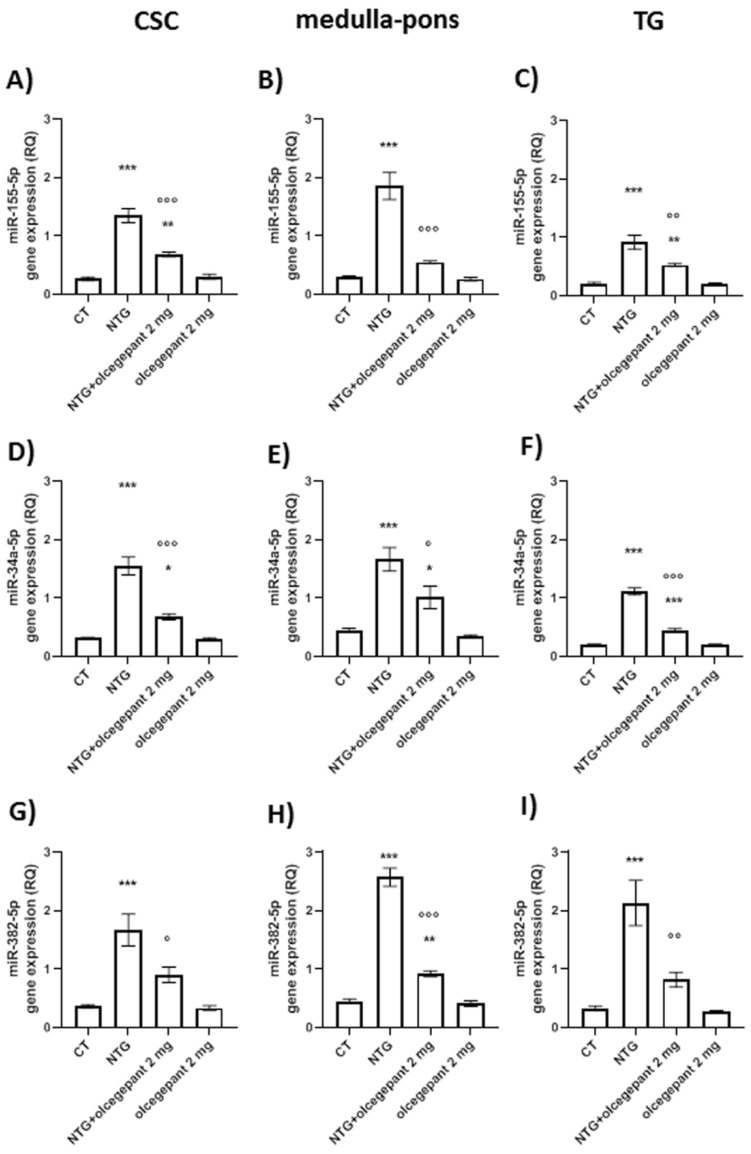
Expression levels of miR-155-5p (**A**–**C**), miR-34a-5p (**D**–**F**), and miR-382-5p (**G**–**I**) in the cervical spinal cord (CSC), medulla–pons, and trigeminal ganglion (TG). microRNA expression is expressed as relative quantification (RQ). Data are expressed as mean ± SEM; one-way ANOVA followed by Tukey’s multiple comparisons test. * *p* < 0.05, ** *p* < 0.01, and *** *p* < 0.001 vs. control (CT) and olcegepant 2 mg; ° *p* < 0.05, °° *p* < 0.01, and °°° *p* < 0.001 vs. nitroglycerin (NTG).

Chronic NTG-treated animals showed a significant increase in miR-155-5p (Figure 7A–C), miR-34a-5p (Figure 7D–F), and miR-382-5p (Figure 7G–I) expression in CSC, medulla–pons, and TG compared to the CT group. These changes were significantly attenuated in all areas by olcegepant treatment (Figure 7). No effect on microRNA levels was observed when olcegepant was given with NTG vehicle (olcegepant 2 mg group) (Figure 7). The findings suggest that CGRP may interfere by still unknown mechanisms in the modulation of inflammatory-related miRNAs.

#### 3.2.5. TRPA1 Gene Expression 

**Figure 8:**
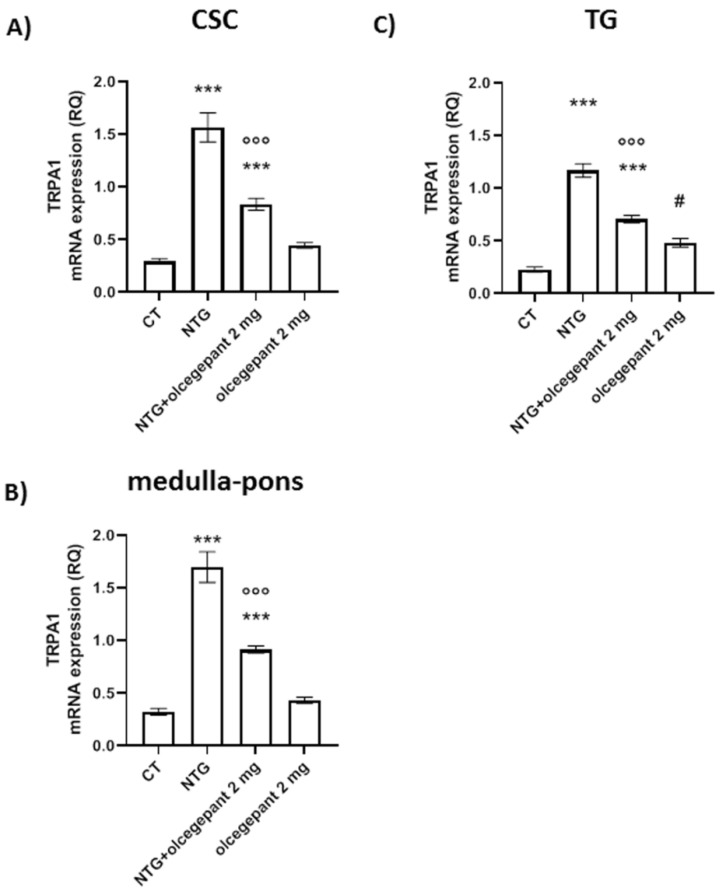
*TRPA1* gene expression levels in (**A**) cervical spinal cord (CSC), (**B**) medulla–pons, and (**C**) trigeminal ganglion (TG). mRNA levels are expressed as relative quantification (RQ). Data are expressed as mean ± SEM; one-way ANOVA followed by Tukey’s multiple comparisons test. *** *p* < 0.001 vs. control (CT) and olcegepant 2 mg; °°° *p* < 0.001 vs. nitroglycerin (NTG); # *p* < 0.05 vs. CT.

Chronic NTG administration caused a significant increase in *TRPA1* mRNA expression levels in all the areas investigated compared to the CT group. These changes were significantly attenuated by olcegepant treatment in all the three areas. Data are reported in Figure 8. These results suggest that the blockade of CGRP receptor, probably by inhibition of pro-inflammatory pathways, interrupts the processes that lead to the neuropeptide release following TRPA1 activation.

## 4. Discussion

In migraine, peripheral and central sensitization play an important pathophysiological role, since they augment pain signal transmission, causing an altered processing of sensory stimuli. In this context, a major role belongs to CGRP, which is expressed in sensory afferents innervating the cranial vasculature and it exerts vasodilatory and neuroinflammatory action, contributing to the development of peripheral and central sensitization.

Here, we explored in depth the interaction of CGRP with other pain mediators relevant for neuronal sensitization in a migraine animal model of chronic migraine, using olcegepant as a pharmacological probe. In this context, our study yielded multiple important pieces of information. First, we confirmed the pivotal role of CGRP in migraine pathophysiology, as suggested by the increase in CGRP gene expression in medulla–pons, CSC, and TGs, as well as in the serum. In addition, we reported that the above-mentioned changes affecting the CGRP pathway are paralleled by an increase in (i) inflammatory cytokines in central and peripheral areas of the nervous system that are relevant in migraine circuitry and in the serum, (ii) miRNA associated to inflammation in the same areas, and (iii) TRPA1 gene expression in the same areas. These effects were all significantly counteracted by olcegepant administration in order to confirm the role of the CGRP pathway.

In agreement, mRNA and protein levels of CGRP were found to be significantly increased in the periaqueductal gray and TNC after NTG administration, supporting an involvement of CGRP in the endogenous pain modulatory system [39,40]. As noted, higher expression levels of CGRP in the brainstem are found in the locus coeruleus [36], a mainly noradrenergic nucleus involved in the regulation of autonomic [41], stress [42], and nociceptive functions [43]. The increase in CGRP serum levels likely reflects a peripherally restricted phenomenon, where CGRP is released from trigeminal fibers outside the blood–brain barrier (dura mater) and drained via intra- and extracranial venous vessels into the jugular blood [44,45,46]. 

Thus, the released CGRP would increase sensory activity at multiple levels, peripheral and central, a feature of migraine pain [47,48]. In the present study, NTG-induced increase in CGRP gene expression was significantly reversed after treatment with olcegepant, as was NTG-induced increase in CGRP serum levels. One of the possible ways through which CGRP is released after NTG is the activation of the TRPA1 channels [49], whose gene expression was increased by the NTG challenge, in agreement with previous data showing that TRPA1 channel activation in trigeminal nociceptive fibers leads to the release of the neuropeptide [12,13]. 

The increase in cytokines observed in the nervous tissues and in the serum in our experiments was inhibited by olcegepant. These findings indicate that the cytokine expression and release is mediated by CGRP. This latter was indeed reported to enhance the expression and release of pro-inflammatory cytokines from human peripheral blood mononuclear cells [8] and lymphocytes [50]. Interestingly, patients with an ongoing migraine attack have higher serum levels of pro-inflammatory cytokines compared to healthy controls, and their serum CGRP levels positively correlate with cytokine’s levels [51]. It is tempting to hypothesize that NTG-activated glial cells have a role in the increased cytokine expression and release in the TGs, CSC, and medulla–pons [52]. In line with this idea, previous data suggest that CGRP stimulates satellite glial cells within the TGs, subsequently to sensory neurons’ sensitization [7,53,54], probably also through the potentiation of the P2Y purinergic receptors [55]. Thus, we can speculate that the blockade of CGRP receptors by olcegepant led to a reduction in CGRP release and, hence, of pro-inflammatory mediators, thus breaking down the pathways that give rise to CGRP release. In line with this hypothesis, olcegepant inhibited TRPA1-mediated dilation [56] and blood flow [57] of the meningeal artery, thus suggesting a reduction of CGRP release [56,57]. Olcegepant-mediated block of pain and inflammation may in turn represent the signal for the system to further reduce CGRP release, again also via TRPA1, thus returning to a physiological level of CGRP receptor stimulation. 

Mounting evidence suggests that several miRNAs expressed in the nervous system play important roles in neuroinflammation and chronic pain [58]. In particular, miR-155 has been shown to be deeply involved in regulating inflammation-associated diseases [5], including migraine [59,60]. Indeed, miR-155 was found to be upregulated in migraine patients in pain-free periods [59]; the same was also reported for miR-382-5p [61]. Intriguingly, the serum upregulation of miR-382-5p is also associated, together with miR-34a-5p, with migraine attacks [61].

In line with the above-mentioned observations, our study shows that the three miRNAs investigated here were upregulated in the model of chronic migraine, while they were downregulated by olcegepant treatment in the central and peripheral areas of the nervous system relevant for migraine pain. This downregulation was associated with a significant reduction of pro-inflammatory cytokines (gene and protein expression) in both peripheral and central nervous system areas, suggesting a relationship between CGRP, miRNAs, and cytokine pathways. 

Recently, Chen et al. [39] reported an increase in TNF-alpha and IL-1beta levels and polarized microglia in TNC after chronic NTG; this suggests the possibility that NTG induces an upstream mechanism in which neuroinflammation is involved [39] and that blockade of the CGRP signaling is able to modulate neuroinflammation and pain. NTG may induce an inflammatory response within the dura mater and TG, potentiated by CGRP release, with an increase in pro-inflammatory cytokines IL-1beta and TNF-alpha levels by nuclear factor kappa B (NF-κB) activation [62,63]. This response may induce an upregulation of miRNAs, not only at the peripheral level but indirectly at the central level, confirming a link with pathways associated with pain transmission/modulation [25]. MiR-382-5p acts as a negative modulator of the interleukin 10 receptor alpha subunit (IL-10RA), an endogenous inhibitor of pro-inflammatory IL-1beta signaling gene expression [61], and thus its reduction is in keeping with the inhibition of CGRP pro-inflammatory activity after olcegepant treatment. Increased miR-382-5p expression in pain conditions may also be related to NF-κB signaling [64], which is a transcription factor for many miRNAs [65], including miR-382 [66]. MiR-34a-5p negatively modulates the GABAergic signaling and thus the reduced expression of miR-34a-5p after olcegepant may reflect a more active GABAergic transmission [61]. In agreement, we previously found that expression of miR-382-5p and miR-34a-5p in peripheral cells of patients with chronic migraine was significantly reduced after erenumab [67], the first-in-class fully human monoclonal antibody targeting the CGRP receptor. Moreover, in another study, we observed that CGRP plasma levels were positively correlated with miR-382-5p and miR-34a-5p in peripheral cells of chronic and episodic subjects, confirming a potential interaction [26] with the CGRP pathway. Here, we also demonstrated a significant upregulation of miR-155-5p, involved in the polarization of microglia and inflammatory processes in a variety of neurological diseases. MiR-155 overexpression promotes a pro-inflammatory phenotype in monocytes/macrophages and triggers the spontaneous production of several pro-inflammatory cytokines, including TNF-alpha, IL-6, and IL-1beta [68,69]. This finding is confirmed by a recent study, where changes in miR-155-5p expression were associated with microglial activation in the TNC after chronic NTG in rats, indicating that miR-155-5p may be involved in trigeminal hyperalgesia [60]. 

**Figure 9:**
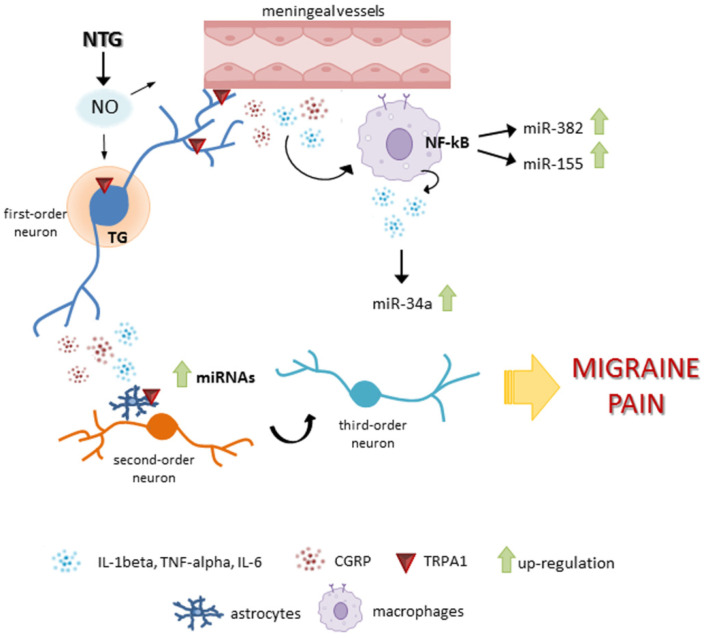
Potential interaction of CGRP with other pain/inflammatory mediators relevant for neuronal sensitization in an animal model of chronic migraine. Nitroglycerin (NTG), by releasing nitric oxide (NO), may induce an inflammatory response within the dura mater and the trigeminal ganglion (TG), potentiated by CGRP release, with an increase in pro-inflammatory cytokines interleukin (IL)-1beta and tumor necrosis factor alpha (TNF-alpha) levels by nuclear factor kappa B (NF-κB) activation [62,63]. In addition, NTG may activate transient receptor potential ankyrin 1 channels (TRPA1) receptors contributing to a further release of CGRP. This response may induce an upregulation of miRNAs, not only at the peripheral level but indirectly at the central level.

Altogether, these observations suggest the possibility that peripheral changes in miRNAs levels, previously reported by us in migraine patients, may likely reflect changes in central/peripheral areas of the nervous system involved in migraine pain modulation. Additionally, the findings also support a role for miR-382-5p, miR-34a-5p, and miR-155-5p in chronic migraine, which is partly, but probably not entirely, linked to the interaction with the CGRP pathway. The potential interplay between CGRP and the inflammatory mediators within the mechanisms of neuronal sensitization is reported in Figure 9.

### Possible Limitations of the Study

A repeated use of acute migraine drugs can induce a condition of medication overuse headache [70]. In light of the experimental settings used in the present study, in which an acute migraine drug was used chronically, it must be noted that we did not detect any change in nociceptive and hyperalgesic behavior in the animals treated chronically with olcegepant and NTG vehicle (Figure 3), nor did we find an increase in the expression of inflammatory cytokines or of CGRP plasma levels, when comparing them with the control group. This is in agreement with previous literature showing that gepants (olcegepant and ubrogepant) did not induce central sensitization upon chronic administration [71,72]. Our data are also in accordance with clinical reports, in which neither gepants nor CGRP-targeting monoclonal antibodies were associated with the development of medication overuse headache [73,74]. 

In this study, we show that chronic CGRP antagonism attenuates NTG-induced trigeminal hyperalgesia. In the clinical setting, olcegepant failed to prevent NTG-induced migraine attacks in subjects with migraine disease [75]. A comparison of methodology between pre-clinical and clinical experiments is of course not possible. However, it is worth noting that our finding is in accordance with a recent study, in which migraine-like intracranial and extracranial neuronal hypersensitivity, as well as central trigeminocervical neurons’ activation stimulated by NTG, were reduced after olgecepant treatment [34]. Additionally, olcegepant was also effective in reducing the NTG-induced plantar allodynia [76] in another pre-clinical study. Another point that must be noted is that, differently from our study in animals and that of Juahsz et al. [45] in humans, Tvedskov and colleagues did not report changes in CGRP levels after NTG in migraine subjects [77].

The findings of the present study suggest a consistent modulation of the biomarkers under investigation in peripheral and central sites, which may be surprising when considering that olcegepant poorly penetrates the blood–brain barrier [78]. Thus, a possible limitation of the present study is that we tested olcegepant following systemic but not intracerebroventricular administration, thus not allowing a more comprehensive analysis of its site of action. It should, however, be noted that in the mouse model of chronic NTG, the intracerebroventricular administration of olcegepant was not effective compared with the systemic administration, thus suggesting that olcegepant has only peripheral effects [79]. Despite this observation, olcegepant was also reported to act in the TNC where it reduced the activation of second order neurons [80]. One possible interpretation is that olcegepant possesses a direct action in the periphery, which subsequently results in an indirect effect at the central level. This effect would in turn modulate the serum levels of the neuropeptide and also its gene expression in peripheral and central areas. An alternative hypothesis is that NTG-associated activation of neuroinflammation and CGRP release may alter blood–brain barrier permeability [81], thus allowing a direct central effect of olcegepant. Specifically targeted studies will be required to confirm or refute these alternative hypotheses.

## 5. Conclusions

The key role of CGRP in migraine pathogenesis and its chronicization is universally accepted. Here, we report that the changes in the CGRP pathway are paralleled by the activation of the neuroinflammation cascade and of miRNAs involved in the inflammatory pathway. To the best of our knowledge, this is the first report to evaluate these downstream molecular mechanisms following ligand-receptor blockade by olcegepant and demonstrating that the CGRP receptor antagonist reduces the mediators of sensitization in peripheral and central areas of the nervous system that are important in the circuitry of migraine pain. 

Altogether, the present data contribute important additional pieces of knowledge in the understanding the complexity of the mechanisms related to CGRP in migraine pathogenesis; it seems reasonable to hypothesize that the miRNAs and cytokines investigated, together with the increased TRPA1 gene expression, may represent only a part of a multi-biomarker panel signature of the migraine disease.

## Figures and Tables

**Table 1:** Experimental groups.

	Experimental Groups	I SET: OFT (*n*)	II SET: rt-PCR; ELISA (*n*)
Acute migraine model	CT (NTG vehicle + olcegepant vehicle)	8	-
	NTG (NTG 10 mg/kg + olcegepant vehicle)	9	-
	NTG + olcegepant 1 mg (NTG 10 mg/kg + olcegepant 1 mg/kg)	6	-
	olcegepant 1 mg (NTG vehicle + olcegepant 1 mg/kg)	6	-
	NTG + olcegepant 2 mg (NTG 10 mg/kg + olcegepant 2 mg/kg)	6	-
	olcegepant 2 mg (NTG vehicle + olcegepant 2 mg/kg)	6	-
Chronic migraine model	CT (NTG vehicle + olcegepant vehicle)	6	6
	NTG (NTG 5 mg/kg + olcegepant vehicle)	6	6
	NTG + olcegepant 2 mg (NTG 5 mg/kg + olcegepant 2 mg/kg)	6	6
	olcegepant 2 mg (NTG vehicle + olcegepant 2 mg/kg)	6	6

CT: control; ELISA: enzyme-linked immunosorbent assay; *n*: number of animals per group; NTG: nitroglycerin; OFT: orofacial formalin test; rt-PCR: real time polymerase chain reaction.

**Table 2:** Primer sequences used in rt-PCR analysis.

Gene	Forward Primer	Reverse Primer
*GAPDH*	AACCTGCCAAGTATGATGAC	GGAGTTGCTGTTGAAGTCA
*TNF-alpha*	CCTCACACTCAGATCATCTTCTC	CGCTTGGTGGTTTGCTAC
*IL-1beta*	TCTTCCTTGTGCAAGTGTCTG	CAGGTCATTCTCCTCACTGTC
*Calca (α-CGRP)*	CAGTCTCAGCTCCAAGTCATC	TTCCAAGGTTGACCTCAAAG
*TRPA1*	CTCCCCGAGTGCATGAAAGT	TGCATATACGCGGGGATGTC
*U6*	TGCGGGTGCTCGCTTCGGCAGC	CCAGTGCAGGGTCCGAGGT
miR-155-5p	TTGAATTCTAACACCTTCGTGGCTACAGAG	TTAGATCTCATTTATCGAGGGAAGGATTG
miR-382-5p	GGCTGTGAGTAATTCTTTGGCAG	GGCAGTATACTTGCTGATTGCT
miR-34a-5p	GCAGTGTCTTAGCTGGTTGTTG	TGCAGCACTTCTAGGGCAGT

*GAPDH*: glyceraldehyde 3-phosphate dehydrogenase; *TNF-alpha*: tumor necrosis factor alpha; *IL-1beta:* interleukin 1beta; *Calca*: calcitonin related polypeptide alpha; *TRPA1*: transient receptor potential ankyrin 1 channels.

## Data Availability

The data presented in this study are available from the ZENODO repository (doi: 10.5281/zenodo.6794106).
